# Relationship between resting augmentation index and spontaneous cardiac baroreflex sensitivity during handgrip exercise in postmenopausal women

**DOI:** 10.1590/1414-431X2024e14152

**Published:** 2025-02-03

**Authors:** E. Prodel, M.F.S.C. Moreira, M.L. Gondim, H.N.M. Rocha, P.A.C. Mira, A.C.L. Nobrega

**Affiliations:** 1 Laboratório de Ciências do Exercício, Departamento de Fisiologia e Farmacologia, Universidade Federal Fluminense, Niterói, RJ, Brasil; 2 Laboratório de Cardiometabologia, Departamento de Fisiologia e Farmacologia, Universidade Federal Fluminense, Niterói, RJ, Brasil; 3 Departamento de Educação Física, Universidade Federal Fluminense, Niterói, RJ, Brasil

**Keywords:** Vascular stiffness, Exercise, Menopause, Blood pressure, Baroreflex

## Abstract

The stiffening of the conductance arteries is a hallmark of ageing and increases drastically after menopause. Therefore, the augmentation index (AIx), a surrogate for arterial stiffness, could be related to the decline in baroreflex sensitivity. We sought to investigate the relationship between resting AIx and spontaneous cardiac baroreflex sensitivity (cBRS) during handgrip exercise in ageing women. Thirteen young women (YW: 24±5 years; 24±2 kg/m^2^) and nine postmenopausal women (PMW: 60±5 years; 26±3 kg/m^2^) underwent the protocol, which consisted of 10 min of supine resting followed by 3 min of static handgrip exercise at 40% of the maximal voluntary force. The AIx was provided by the aortic pressure waveform and cBRS was calculated using the sequence technique, and vagal activity was accessed via heart rate variability using the root mean square of successive differences (RMSSD) index. Resting AIx was higher in PMW compared to YW (YW: 8±10%; PMW: 23±8%; P<0.01), while the cBRS (YW: 16±12 ms/mmHg; PMW: 10±5 ms/mmHg; P=0.08) and RMSSD (YW: 46±35 ms; PMW: 34±12 ms; P=0.26) were similar in YW and PMW. At rest, there was no significant (P>0.05) relationship between the AIx and cBRS in YW and PMW. However, in PMW, a negative (slope=-0.22) and strong (r=-0.70; P=0.03) relationship was observed between AIx and cBRS for the increment of blood pressure during the handgrip exercise. The stiffening of the arterial tree is one possible mechanism to explain the decrease of spontaneous cardiac baroreflex sensitivity during exercise in postmenopausal women.

## Introduction

Healthy ageing, which is related to the decline of physiological functions, is a physiological process shared by all living beings (1). In women, ageing is marked by menopause, when the production and release of ovarian hormones (i.e., estrogen and progesterone) decrease (2). Interactions between the ovarian hormones and the vascular smooth muscle could explain vascular ageing after menopause since the ovarian hormones play an important role in modulating cardiovascular responses (3). The stiffening of the conductance arteries, which is related to chronic hypertension, increased cardiac work, and higher cardiovascular risk, is considered a hallmark of ageing and increases drastically in postmenopausal women (4-6).

The acute increase in blood pressure stretches the peripheral baroreceptors (e.g., aortic arch and carotid bifurcation) and depolarizes the afferent nerve to the cardiovascular centers in the medulla oblongata (e.g., the nucleus of the solitary tract [NST]), causing reflex-mediated efferents to the heart via the vagal nerve and increasing the length of RR intervals (7,8). In stiffened arteries, compared to compliant arteries, each unit of increase in blood pressure elicits a reduced afferent discharge from the peripheral baroreceptors to the NST. Consequently, there is a decreased vagal efferent feedback to the heart and cardiac baroreflex sensitivity (cBRS) (9).

Several mechanisms control the cardiovascular changes during exercise, including the mechanoreceptors, metaboreceptors, and the central command (10). Specifically, the central command evokes a resetting of the carotid baroreceptors, which work at higher levels of blood pressure during exercise (11). The sensitivity of the cardiac baroreflex in physiological conditions seems to be preserved during exercise (12). However, the spontaneous cardiac baroreflex sensitivity during exercise and its possible relationship with the increase of arterial stiffness accompanied by menopause has not been fully investigated. Reduced cBRS in postmenopausal women is related to higher transient changes in blood pressure during vasodilatory stimulus (13). Additionally, reduced cBRS is associated with cardiometabolic risks and prehypertension status after menopause (14). The decrease in the sensitivity of the reflex-related response to the increase in blood pressure could evoke an exaggerated increase in blood pressure during exercise increasing cardiovascular risk.

Therefore, we sought to investigate the relationship between resting augmentation index (AIx) and cBRS during handgrip exercise in postmenopausal women. The hypothesis was that the increased AIx is related to a decreased sensitivity of the arterial baroreceptors during static handgrip exercise.

Handgrip static exercise was used as a research tool because it produces small increases in heart rate and robust increases in blood pressure compared to dynamic exercises (10). Arterial stiffness can be measured non-invasively using applanation tonometry, and AIx can be used as a surrogate for arterial stiffness, where higher AIx indicates higher arterial stiffness (15-17). Further, cBRS can be assessed using spontaneous variation in systolic blood pressure accompanied by synchronic changes in R-R intervals (18).

## Material and Methods

### Participants

All experimental procedures were approved by the Ethics Committee of the Fluminense Federal University Antonio Pedro University Hospital (#2.362.515) and conducted according to the Declaration of Helsinki. The study was registered in a clinical database (19). After receiving detailed verbal and written information about the protocol, each participant signed a consent form. Thirteen young women and nine postmenopausal women were included. Eligibility criteria for participation were apparently healthy women with no history or symptoms of chronic diseases and who were physically active. The protocol was conducted before the COVID-19 pandemic. Young women (YW) were tested during the early follicular phase of the menstrual cycle or during the low-hormone phase in women using oral contraceptives (n=5) to minimize the acute cardiovascular effects of reproductive hormone variation across the menstrual cycle (20). Additionally, young women had to have not breastfed for at least two years (21). Menopause was defined as at least one year since the last menstruation (2) and postmenopausal women (PMW) must have never received hormonal replacement therapy. All participants were regularly engaged in low-intensity recreational physical activity. Participants were asked to abstain from caffeine, alcohol, and exercise 24 h before the experimental session, which was conducted 2 h after eating a light meal (17).

### Experimental measurements

Heart rate (HR) was continuously monitored using a standard lead II electrocardiogram (Powerlab; ADInstruments, Australia), and beat-to-beat blood pressure (BP) was measured by photoplethysmography in the middle finger of the left hand (Finometer Pro; Finapres Medical Systems, Netherlands). The data were sampled at 1000 Hz. Brachial blood pressure was obtained from the brachial artery of the left arm using the oscillometric method (Omron Dalian Co., HEM-7113, Japan).

Applanation tonometry was used to acquire the radial pulse waveform during the 10 min of resting (SphygmoCor System; AtCor Medical, USA). To assess central hemodynamics, a validated generalized transfer function was applied on the left radial artery pulse waveforms calibrated by left brachial systolic and diastolic BP before every tonometric measure of the radial pulse waveform (22). The pulse wave analysis of the aortic pressure waveform provided the augmentation pressure [Aug (mmHg)], AIx (%), and the AIx adjusted for heart rate at 75 beats per minute [AIx at 75 bpm (%)] (15-17).

cBRS (ms/mmHg) was calculated using the sequence technique, which consists of the identification of progressive increases or decreases in systolic blood pressure (SBP) followed by a progressive enlargement or shortening of RR intervals, respectively. The sequences (>3) were considered only when spontaneous fluctuations in SBP and RR interval were ≥1 mmHg and ≥1.0 ms. Linear regression was applied to each sequence, and the ones in which the “r” was >0.85 were considered (CardioSeries v2.4, Brazil) (18). The sequence technique assumes that successive spontaneous increases or decreases in systolic blood pressure elicit baroreflex-mediated fluctuations in the RR interval length (18). For individuals with higher cBRS, the shorting of the RR intervals for each mmHg of increase in blood pressure is higher. cBRS was calculated during the 10-min rest and during the three minutes of handgrip exercise for sequences of increase in blood pressure (18).

Short-term vagal modulation was accessed via heart rate variability (HRV) using the root mean square of successive differences in the standard deviation of the R-R intervals (RMSSD) (23,24). Briefly, the R-R intervals were extracted from the ECG signal during the 10-min rest (baseline) and during the three minutes of handgrip exercise. After that, the RMSSD was calculated in the time domain from the R-R series (Kubius, Finland) (25).

### Experimental protocol

The experimental protocol was conducted during one laboratory visit. The experimental visit consisted of 10-min rest followed by 3 min of static handgrip exercise at 40% of the maximal voluntary force, which was previously measured. The room was maintained at a controlled temperature between 22 and 24°C. For the duration of each experimental visit, participants were tested in a semi-recumbent position with a backrest support set at a 45° angle to the bed. After instrumentation, an electronic handgrip dynamometer was used to determine the maximal voluntary force as an average of 3 maximal efforts 1 min apart, which did not differ by over 5% (Powerlab, AD Instruments, New Zealand). Participants then sat quietly for 10 min until all variables were stable. Then a resting baseline period of 10 min was followed by static handgrip exercise at 40% of the maximal voluntary force for 3 min. The handgrip exercise was performed with the dominant hand and all participants had right-hand dominance (17). Ratings of perceived exertion during the handgrip exercise were obtained using the modified 1-10 Borg scale (26,27). All cardiovascular variables were sampled at 1000 Hz (LabChart v8.0 and Powerlab, AD Instruments).

### Statistical analyses

Data are reported as means±SD and median and interquartile interval for RMSSD. A two-way analysis of variance was used to test the differences between young and postmenopausal (Group) women during rest and exercise (Condition) and their interactions. The relationship between AIx and cBRS was evaluated using the Pearson correlation coefficient. Data distribution was tested by the Kolmogorov-Smirnov test. Participant characteristics and cardiovascular hemodynamics at rest were compared using a *t*-test or U-test when the variables were not normally distributed. Statistical significance was set at P≤0.05. All analyses were performed using SPSS (v.26, IBM, USA) and graphs were created using GraphPad Prism 6 software (USA).

## Results

Participant characteristics are reported in Table 1. Young and postmenopausal women had similar weight, height, and body mass index. HR increased during exercise in young and postmenopausal women, but a higher increase was observed in young women (YW: Δ22±9 bpm; PMW: Δ11±6 bpm; P=0.01). Handgrip exercise increased SBP (YW: Δ40±13 mmHg; PMW: Δ46±25 mmHg; P<0.01 for time effect) and DBP (YW: Δ27±8 mmHg; PMW: Δ23±14 mmHg; P<0.01 for time effect) similarly in both groups.

**Table 1:** Characteristics of young women and post-menopausal women participating in the study.

	Young (n=13)	Postmenopausal (n=9)	P*-*value
Age (years)	24±5	60±5	**<0.01**
Weight (kg)	64±9	66±5	0.48
Height (cm)	163±6	160±3	0.30
BMI (kg/m^2^)	24±2	26±3	0.12
MVC (N)	257±77	216±57	0.18

Data are reported as means±SD. BMI: body mass index; MVC: maximal voluntary contraction. The *t*-test was used for analyses with the statistical significance set to P≤0.05. P-values in bold type indicate statistically significant.

**Figure 1:**
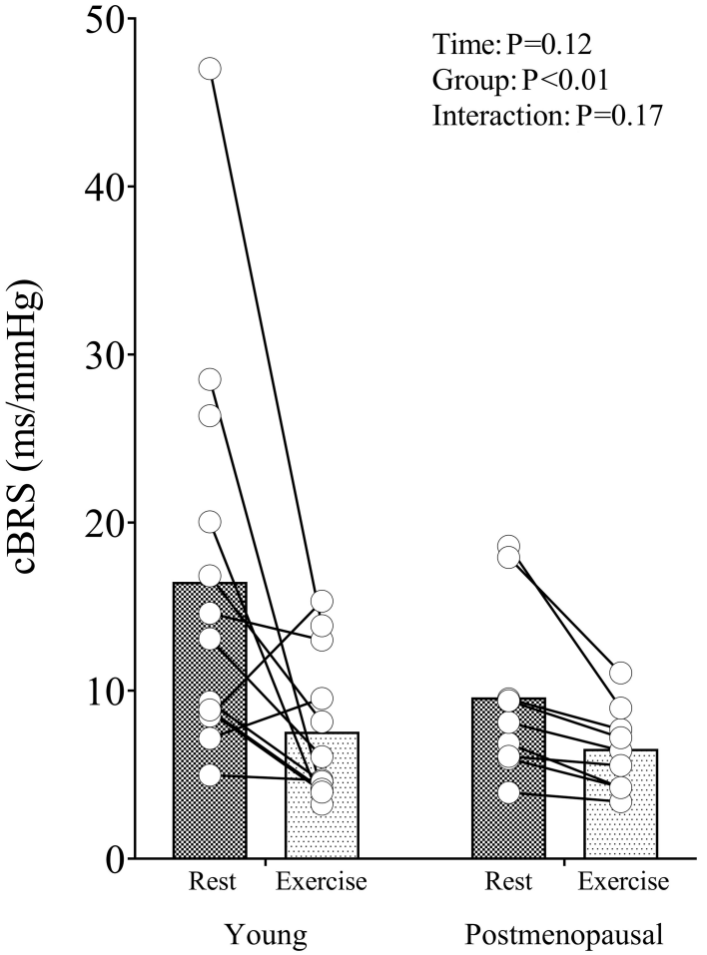
Spontaneous cardiac baroreflex sensitivity (cBRS) at rest and during handgrip exercise of young women and post-menopausal women. The box represents the mean value. Two-way ANOVA was used for analyses with the statistical significance set to P≤0.05.

Spontaneous cBRS was similar in young and postmenopausal women at rest (Table 2), and the handgrip exercise did not change spontaneous cBRS in young and postmenopausal women (YW: Δ-10±11 ms/mmHg; P<0.01; PMW: Δ-4±5 ms/mmHg; P=0.50, Figure 1). Resting Aug and AIx were higher in postmenopausal women compared to young women (Table 2).

**Table 2:** Cardiovascular hemodynamics of young women and post-menopausal women at rest.

	Young (n=13)	Postmenopausal (n=9)	P*-*value
HR (bpm)	72±8	65±7	**0.04**
SBP (mmHg)	104±7	114±14	0.13
DBP (mmHg)	68±5	71±8	0.75
MAP (mmHg)	80±5	86±10	0.32
Aug (mmHg)	1.5±2.4	10.0±4.8	**<0.01**
AIx (%)	8±11	27±7	**<0.01**
AIx at 75 bpm (%)	8±10	23±8	**<0.01**
cBRS (ms/mmHg)	16±12	10±5	0.08
RMSSD (ms)	32 [21-57]	36 [25-45]	0.55

Data are reported as means±SD. HR: Heart rate; SBP: systolic blood pressure; DBP: diastolic blood pressure; MAP: mean arterial pressure; Aug: augmentation pressure; Alx: augmentation index adjusted for 75 heart beats per minute; cBRS: cardiac baroreflex sensitivity; RMSSD: root mean square of successive differences in the standard deviation of the R-R intervals reported as median and interquartile range. The *t*-test or U-test were used for analyses with the statistical significance set to P≤0.05. P-values in bold type indicate statistically significant.

At rest, RMSSD was similar between groups and decreased equally during handgrip exercise in young and postmenopausal women (YW: Δ-28±5 ms; P<0.01; PMW: Δ-22±14 ms; P<0.01 for time effect).

**Figure 2:**
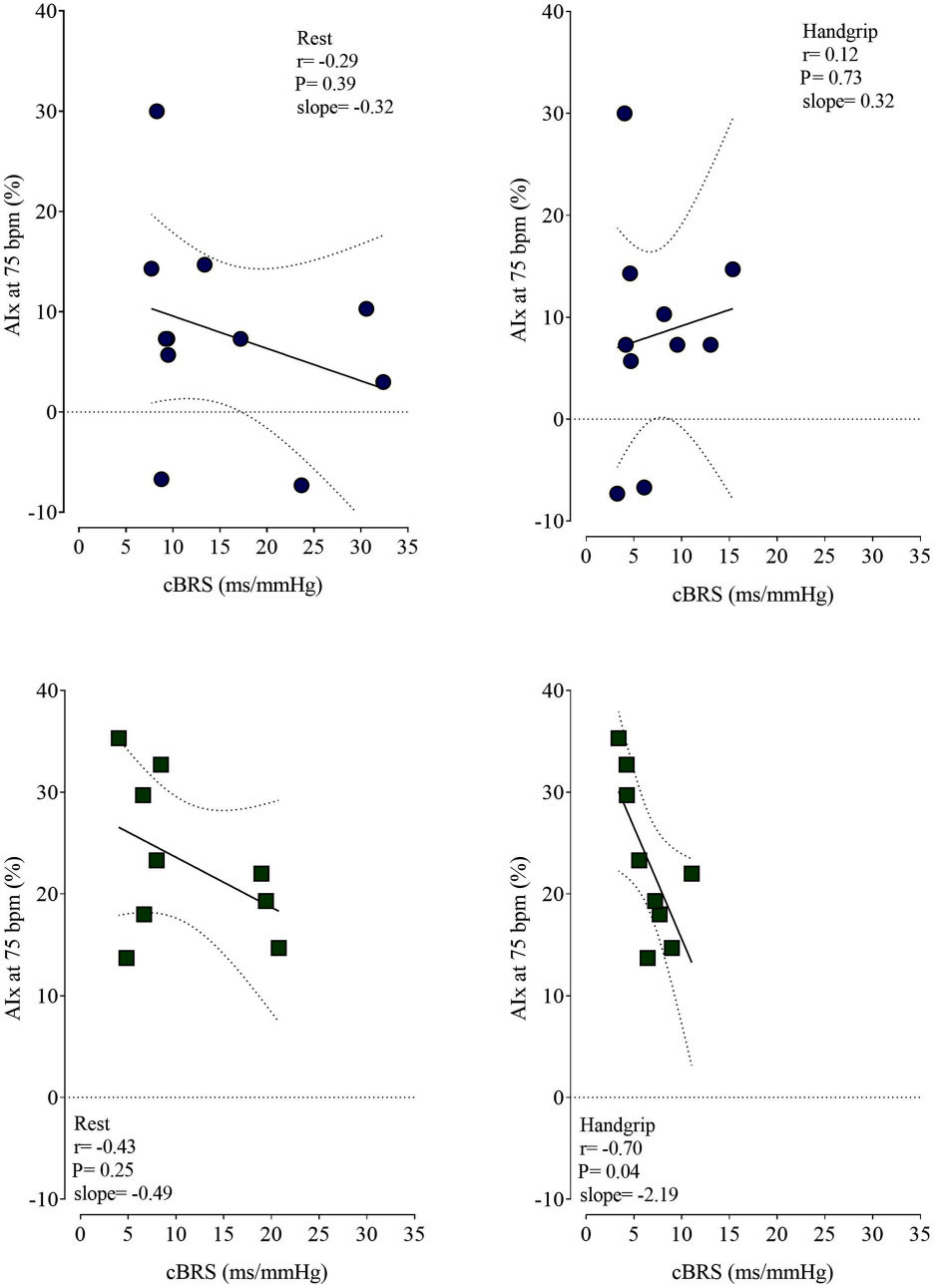
Relationship between arterial stiffness and cardiac baroreflex sensitivity. The black lines are the regression lines, and the dotted lines are the confidence intervals of the association between augmentation index (AIx) adjusted for 75 heartbeats per minute and cardiac baroreflex sensitivity (cBRS). Blue circles represent young women, and green squares represent postmenopausal women. Pearson correlation was used for analyses with the statistical significance set to P≤0.05.

At rest, there was no relationship between AIx and baroreflex sensitivity in young and postmenopausal women, but during the handgrip exercise, a negative (slope=-0.22) and strong (r=0.70; P=0.03) relationship between AIx and cBRS for the increment of blood pressure was observed in postmenopausal women (Figure 2).

## Discussion

The crucial finding of this study was that the resting augmentation index in postmenopausal women was related to decreased spontaneous cardiac baroreflex sensitivity during handgrip exercise.

Ageing is a complex process leading to progressive structural and functional physiological decline. In women, the loss of the ovarian hormones (i.e., estrogen and progesterone) accelerates vascular ageing. Estrogen affects the modulation of the autonomic nervous system. In young women, vagal modulation, assessed by heart rate variability, seems to be lower during the follicular phase of the menstrual cycle (28). Several studies show a reduced vagal modulation after menopause (28) and our results confirmed those studies when the women were at rest. Exercise induces vagal withdrawal and sympathetic activation related to the exercise intensity, marked by decreased heart rate variability (29). In our study heart rate variability decreased during handgrip exercise only in young women. In older men, heart rate variability seems to decrease during leg static exercise (30). However, further studies in women are fundamental to confirm this finding and understand the physiological mechanisms underlying that response.

During menopause, vascular health is affected, which might explain the increased cardiovascular risk in postmenopausal women compared to younger women and aged-matched men (31). The intima-medial thickness increases with ageing in men and women, but before menopause, women present a slight increase in intima-medial thickness compared to men at the same age. After menopause, the increase rate in intima-medial thickness is much higher in women (4). The thickening of the arterial wall increases arterial stiffness, increasing pulse wave conductance velocity and consequently increasing pressure during the systolic phase of the cardiac cycle. The pulse amplification during systole is related to several vascular risks observed in ageing, including hypertension (6). In our study, postmenopausal women had higher AIx, which seems to be related to physiological ageing. Nevertheless, resting blood pressure was within normal range. Although the loss of estrogens might be an important component of the changes in cardiovascular function in postmenopausal women, we must consider the ageing process itself.

The cardiac baroreflex is a fundamental mechanism for the beat-to-beat control of blood pressure (31), which declines with ageing. In healthy older men, the decline of cardiac baroreflex sensitivity seems to be independently associated with aortic stiffening (9). This could occur because the baroreceptors are located at the central arteries and this stiffening reduces the response to the acute increase in blood pressure. Consequently, the stretch of the baroreceptors for each unit of increase in blood pressure would be diminished when compared to complacent arteries (8). Interestingly, the incidence of hypertension in young women is lower than in men of the same age, but higher in postmenopausal when compared to age-matched men (31).

During exercise, the baroreflex regulates blood pressure at higher levels (i.e., baroreflex resetting), and a physiological baroreflex control is necessary for an appropriate exercise-related blood pressure response (12). Impaired baroreflex sensitivity could evoke altered cardiovascular responses during exercise. In our study, AIx, a surrogate for arterial stiffness, negatively correlated with cBRS in postmenopausal women, therefore the stiffening of the arterial tree seems to be one of the mechanisms to explain the decrease in cBRS. However, an exaggerated arterial pressure response during exercise was not observed in postmenopausal women compared to young women in this study. One possible explanation is that, although the participants were not engaged in high-intensity training, all were physically active. It is known that physical exercise protects the cardiovascular system (32).

For the future, it is important to investigate the mechanisms behind reduced cBRS in postmenopausal women at rest and during exercise. In our study a correlation was shown, but this does not suggest causality, so it could be two independent effects of menopause and aging. Furthermore, the effect of acute exercise on autonomic modulation is not fully understood in women after menopause. Although experimentally difficult, the effect of menopause separated from the effect of aging should be addressed.

### Experimental considerations

Limitations in this study should be considered. We used a generalized transfer function to indirectly assess arterial stiffness, but previous studies showed this to be a reliable tool (22,33-35). The baroreflex was assessed via the sequence method, which reaches a limited range of pressure change, but still better mimics real-life physiological blood pressure changes. We tested young women using or not using oral contraceptives. Although we cannot exclude a possible effect, a plethora of studies show no effect of contraceptive use on autonomic modulation (28). We tested only healthy active women, and the results should not be extrapolated to other populations. We used static handgrip exercise, in which only a small muscle mass is used. Therefore, different results might be achieved with dynamic exercises that use large muscle mass (e.g., cycling, running, and swimming). This was a small study and we only tested nine postmenopausal women. The reason was that it is very difficult to recruit women that are physically active, healthy, and have never taken hormone replacement therapy.

## Conclusion

Our results led us to conclude that the stiffening of the arterial tree is one possible mechanism to explain the decrease of spontaneous cardiac baroreflex sensitivity during exercise in postmenopausal women.
